# New parameters on prediction of severity of transient tachypnea of the newborn

**DOI:** 10.55730/1300-0144.5402

**Published:** 2022-04-10

**Authors:** Yusuf ÇELİK, Dilek KAHVECİOĞLU, İbrahim ECE, Fatih ATİK, Aslıhan KÖSE ÇETİNKAYA, Medine Ayşin TAŞAR

**Affiliations:** 1 Department of Pediatrics, Nafiz Körez State Hospital, Ankara, Turkey; 2 Division of Neonatology, Department of Pediatrics, University of Health Sciences, Ankara Training and Research Hospital, Ankara, Turkey; 3 Division of Pediatric Cardiology, Department of Pediatrics, University of Health Sciences, Ankara City Hospital, Ankara, Turkey; 4 Division of Pediatric Cardiology, Department of Pediatrics, Liv Hospital, İstanbul, Turkey; 5 Department of Pediatrics, University of Health Sciences, Ankara Training and Research Hospital, Ankara, Turkey

**Keywords:** Transient tachypnea of the newborn, nucleated red blood cells, pulmonary hypertension

## Abstract

**Background/aim:**

Transient tachypnea of the newborn (TTN) is a common clinical problem that often occurs in the first hours of life. Although it is considered to be a benign clinical course, some cases may have severe symptoms and require ventilation support. In this study, we aimed to determine the association between the mean platelet volume (MPV), nucleated red blood cells (NRBCs), right ventricular systolic pressure (RVSP), and the severity of TTN.

**Materials and methods:**

Patients with TTN were divided into two groups according to Silverman score (<7: group 1 [n: 34] and ≥7: Group 2 [n: 30]). The groups were compared in terms of demographic characteristics, hematologic parameters, and RVSP within the first 24 hours after admission.

**Results:**

Mean birth weight of the patients was 3033.4 **±** 364.1 g and median gestational age was 38 weeks (min–max: 34–42). Patients in Group 2 were found to require higher nasal continuous positive airway pressure (nCPAP) support and longer duration of oxygen treatment (p: 0.001). Patients in Group 2 had significantly higher thrombocyte, absolute NRBCs count, NRBCs/100 WBCs, and RVSP levels (p < 0.05). Hemoglobin and hematocrit levels were found significantly higher in group 1(p < 0.05). In logistic regression analysis, NRBCs/100 WBCs was found to be the most important independent parameter that affects Silverman score at admission (OR: 7.065, CI: 1.258–39.670, p: 0.026).

**Conclusion:**

This is the first study that investigates the association between NRBCs, RVSP, and severity of TTN. We think that elevated NRBCs and RVSP values are helpful for clinicians in decision making for referral of the patients to a secondary or a tertiary level of NICU and also inform the families about prognosis.

## 1. Introduction

Delayed resorption of fetal lung fluid is thought to be the underlying cause of transient tachypnea of the newborn (TTN) [1]. Birth by cesarean section (C/S), maternal diabetes and asthma, birth without labor, lower gestational age, macrosomia, male sex, and perinatal asphyxia are common risk factors for TTN [2–4]. TTN is a benign disorder in which clinical symptoms generally regress spontaneously within 48–72 hours.

Nucleated red blood cells (NRBCs) increase in response to hypoxia [5,6]. Elevated NRBC counts found to be in relation with morbidity and mortality in perinatal asphyxia and preterm infants [5–8]. Mean platelet volume (MPV) is a marker of thrombocyte activation and it is elevated in some inflammatory conditions. MPV values in respiratory distress syndrome (RDS) and neonatal pneumonia were found to be higher and have a relation with severe clinical conditions [9,10].

There has not been a specific treatment for TTN; adequate oxygenation and nutrition support are generally sufficient for follow-up [11]. Although it generally has a good prognosis, clinical conditions may deteriorate because of pulmonary congestion. Vasoconstriction of pulmonary vessels and increase in pulmonary vascular resistance will result in the most feared complication as persistent pulmonary hypertension (PPHT) [12].

We hypothesize that MPV, NRBC, and right ventricular systolic pressure (RVSP) values will be higher in patients with TTN who have high respiratory scores at admission and these patients will need more respiratory support. We think that elevated MPV, NRBC, and RVSP values could be helpful for clinicians in decision making for referral of the patients to a secondary or a tertiary level of NICU and also inform the families about prognosis.

## 2. Materials and methods

This prospective single-center study included 64 patients admitted to the University of Health Sciences Ankara Training and Research Hospital Neonatal Intensive Care Unit between December 2019 and May 2020.

During the study period, 106 infants with gestation week ≥34 weeks with any respiratory symptoms were evaluated. Among the 106 infants, 42 were excluded (Figure).

### 2.1. Selection of study population

Infants at ≥34 weeks of gestation age with any respiratory symptoms (tachypnea, retraction, grunting, cyanosis) that continued more than 6 h after birth were evaluated for differential diagnosis of TTN.

Clinical and radiological findings were used for the diagnosis of TTN [4].

1. Respiratory distress including tachypnea, retractions, grunting, cyanosis, and nasal flaring starting within 6 h after birth and persisting ≥12 hours.

2. The presence of at least one of the following findings in chest X-ray: prominent pulmonary vascular markings, symmetrical perihilar congestion, hyperinflation of the lungs, and fluid in the interlobar fissures.

3. Exclusion of other diagnosis that result in respiratory distress such as surfactant deficiency, congenital pneumonia, meconium aspiration syndrome, congenital heart disease or metabolic disorder.

### 2.2. Exclusion criteria

1. Patients with < 34 weeks of gestational age.

2. Patients whose symptoms of respiratory distress continued less than 6 h (transition delay).

3. Patients with congenital heart disease, asphyxia (pH < 7.0 and BE > −12), RDS, meconium aspiration syndrome, and metabolic disorders like hypoglycemia and polycythemia.

4. Patients with major congenital and/or chromosomal anomaly.

5. Patients lacking informed consent form from patients.

6. Patients with cardiological anomalies.

7. Patients who could not be assessed with echocardiography were excluded from the study.

Patients with TTN were divided into two groups according to Silverman score (<7: group 1 [n: 34] and ≥7: Group 2 [n: 30]) (Figure). The groups were compared in terms of sex, gestation week, birth weight, delivery method, mother’s age, APGAR scores at the 1st and 5th min, respiratory rate at admission, clinical findings at the time of admission, duration of oxygen support, duration of noninvasive and invasive ventilation support, and duration of hospitalization.

The groups were compared in terms of hematological parameters (white blood cells (WBC), hemoglobin (Hgb), hematocrit (Hct), MPV, platelet, RDW, absolute NRBC count, and NRBCs/100 WBCs) obtained at admission. At least 1 cc blood collected into ethylene diamine tetra acetic acid (EDTA) tubes complete blood count (CBC) were analyzed with an automated hematology analyzer (ADVIA; 2120 Siemens Healthcare Diagnostics).

Transthoracic echocardiographic evaluation was performed with Philips EPIQ device and 6–10 MHz probe. Tricuspid regurgitation flow velocity was measured in 3 consecutive cycles by Doppler evaluation, and estimated RVSP value was obtained by adding estimated right atrial pressure (5 mmHg) to the value obtained by Bernoulli’s equation. Also the groups were compared in terms of pulmonary pressure measured with echocardiography that was performed bedside by a pediatric cardiologist in the first 24 h after admission. RVSP was calculated and assessed via peak tricuspid flow velocity in order to detect the increase in patients’ pulmonary pressure. Mean pulmonary artery pressure could not be measured directly as pulmonary valve insufficiency could not be diagnosed in the patients.

In order to assess the patients’ respiratory system findings, modified Silverman score (chest movement, subcostal and xiphoid retractions, nasal flaring, and grunting) and modified Downes score (respiration rate, cyanosis, retraction, grunting, and air entry) were used [13–15].

### 2.3. Ethics committee approval

This study was conducted under the approval (dated 28.11.2019 and numbered 104/2019) of the University of Health Sciences Ankara Training and Research Hospital Clinical Research Ethical Committee. Oral and written consent was taken from the families that accepted to participate in the study.

### 2.4. Statistical analysis

Statistical analysis was performed using SPSS version 16.0 (SPSS, Chicago, IL, USA). To compare the proportions among the groups, either χ2 test or Fisher’s exact test was used; to compare the means among the groups the t-test or the Mann–Whitney *U* test was used depending on the sample size. p-value of <0.05 was regarded as statistically significant. Logistic regression analysis was performed to assess independent variables that affected the Silverman score at admission in multivariable analysis. The Hosmer–Lemeshow test was used for model fit. The power level desired was 0.80, and consequently, 30 patients were needed for each group.

## 3. Results

Sixty-four newborns included in the study were divided into two groups according to the Silverman score evaluated at admission. When the groups were compared in terms of demographical features, C/S was found to be higher (p = 0.015), and 1st and 5th min APGAR scores (p = 0.001, p = 0.003, respectively) were found to be lower in Group 2 (Table 1).

When the groups were compared according to respiratory findings: respiratory rate at admission, Downes score at admission and at 24th h, Silverman score at 24th h, and duration of oxygen treatment and hospitalization were significantly different between groups (Table 2). In Group 2, there were 3 patients who were intubated; Downes score at 24th h were 6, 5, 9 and Silverman score at 24th h were 7, 6, 10, respectively, of these 3 patients.

Times of hemogram sample collection were 2.6 ± 1.8 and 2.8 ± 2 h in group 1 and 2, respectively (p = 0.61). When the groups were compared in terms of hematologic parameters; number of platelets, absolute NRBC count, and NRBCs/100 WBCs were significantly higher in Group 2 (p: 0.001). RVSP values were found to be higher in Group 2 (p = 0.001) (Table 3).

Assessment of the aforementioned significantly different parameters on logistic regression demonstrated NRBCs/100 WBCs independently had an odds ratio of 7.06 (95% CI: 1.25–39.6) for assessing severity of TTN (Table 4).

## 4. Discussion

In this prospective cohort study, we demonstrated that higher NRBCs counts at admission and RVSP values at 24 h were associated with the severity of TTN.

TTN is a benign and self-limited condition; however, patients may deteriorate because of the hypoxia and respiratory failure [1]. TTN occurs due to disruption of clearance of lung fluid and when delivery occurs before the onset of spontaneous labor, neonatal transition will be impaired [14]. In accordance with the literature, in our study, rate of C/S was 47.1% in Group 1 and 76.7% in Group 2 (p: 0.015). Based on our findings, it is considered that decreasing the rate of elective C/S would prevent morbidities like TTN.

Megakaryocytes are precursors of thrombocytes. After megakaryocytes are produced in bone marrow, a substantial part of megakaryocytes reaches the lungs and produces thrombocytes. Damages caused by hypoxia in lungs may suppress thrombocyte production [16]. Besides, thrombocytosis occurs as a result of interleukin production caused by infection and inflammation [17]. İlhan et al. evaluated 101 patients with TTN, who were divided into two groups according to requirement of respiratory support. As a result of the study, they found that lower thrombocyte counts were associated with longer support duration [18]. However, in our study, thrombocyte counts were founded to be higher in patients with more severe clinical findings (p: 0.016). Elevated thrombocyte levels occur as a result of stimulation of megakaryopoiesis due to inflammatory conditions [19]. Our findings may be attributed to this mechanism.

MPV is a parameter of platelet activation. It could be elevated during the inflammation [20]. Canpolat et al. found that MPV elevated in response to increased platelet consumption due to lung damage in RDS [10]. In a study conducted by Sakurai et al. on 68 newborns, MPV values were found to be higher in patients who required invasive ventilation support [21]. In our study, no significant difference was found between the groups in terms of MPV values (p > 0.05). These findings could be attributed to milder lung damage of our patients and less invasive ventilation support.

NRBCs were produced in bone marrow as precursors of erythrocytes and increase in response to erythropoietin due to hypoxic conditions in newborns. In a study conducted by Boskabadi et al. on 63 patients with perinatal asphyxia, NRBC counts were found to be significantly higher in the patients who had severe clinical conditions and developed complications [22]. Çığrı et al. found that the NRBC levels were higher in infants with TTN compared with infants in the control group, but they did not mention the association between NRBCs and the severity of TTN [23]. In our study, NRBCs/100 WBCs and absolute NRBC counts were found to be significantly higher in the group with higher Silverman scores (p: 0.001). In Logistic regression analysis, NRBCs/100 WBCs was determined to be the most effective independent parameter that affects Silverman scores (OR: 7.065, CI: 1.258–39.670, p: 0.026). This study is unique in that it evaluated the association between NRBCs and the severity of TTN. And this marker is considered to be a prognostic marker for TTN.

Silverman score is a scoring system that shows the degree of clinical severity in newborns who are admitted to the hospital with respiratory distress [13]. Kahvecioğlu et al. evaluated 35 patients with TTN, Silverman scores were found to be significantly higher in the group that received CPAP and mechanical ventilation than the group that received O_2_ treatment [24]. In a study conducted by Headstorm et al. on 140 newborns with respiratory distress, the patients with higher Silverman scores were found to have longer duration of ventilation support, longer duration of hospitalization, and more invasive ventilation support [13]. Like previous studies, we found that the patients with higher Silverman score had longer duration of ventilation support, longer duration of hospitalization, and need of more invasive and noninvasive ventilation. In light of these findings, we think that using Silverman score would be useful in terms of predicting patients’ prognosis, duration of hospitalization, and need of ventilation support.

In prenatal period, merely 1% of the blood entering the heart transmits to lungs. Following the birth, the amount of the blood reaching the lungs increases as a result of the decrease in vascular resistance in lungs depending on vasodilation. Pulmonary resistance may increase in lung diseases like TTN and RDS, and it may result in pulmonary hypertension [25,26]. In the diagnosis of pulmonary hypertension, the gold standard test is the measurement of pulmonary artery pressure via right heart catheterization. On the other hand, instead of invasive catheterization, RVSP measurement over tricuspid valve regurgitation flow can be used as a noninvasive and reliable method in newborns [27]. Vyas-Read et al. determined the relationship between RVSP and mortality in infants with severe BPD and found out that RVSP values above 40 mmHg significantly increased mortality [28]. The present study is the first study that compares severity of TTN and RVSP. In our study, RVSP values of the cases in Group 2 were found to be significantly higher (p: 0.001). In logistic regression analysis, RVSP levels were determined to be one of the independent parameters that affect the Silverman score at admission (OR: 1.2, CI: 1.030–1.399, p: 0.02). In light of these data, it may be considered that high RVSP values measured at 24th h may be associated with poor prognosis and RVSP may be used as an indicator of severity of TTN.

Our study has some limitations; it is a single-center study with limited number of cases and during the study period, all the patients diagnosed with TTN could not be included in the study. It is known that NRBCs increase in association with hypoxia. Although patients with hypoxic ischemic encephalopathy were excluded in our study, both groups could not be compared in terms of pH, pO_2_, and lactate levels due to lack of data. It is expected that RVSP will decrease after delivery, but due to technical difficulties, repeated RVSP measurements of the patients could not be performed.

In conclusion, to the best of our knowledge, our study is the first study that investigates the relationship between NRBCs, RVSP, and the severity of TTN. The present study showed that the patients whose NRBCs at admission and RVSP values at the first 24 h were higher had poor prognosis and needed more ventilation support and longer duration of hospitalization. We think that elevated NRBC and RVSP values are helpful for clinicians in decision making for referral of the patients to a secondary or a tertiary level of NICU and also inform the families about prognosis.

## Figures and Tables

**Figure:**
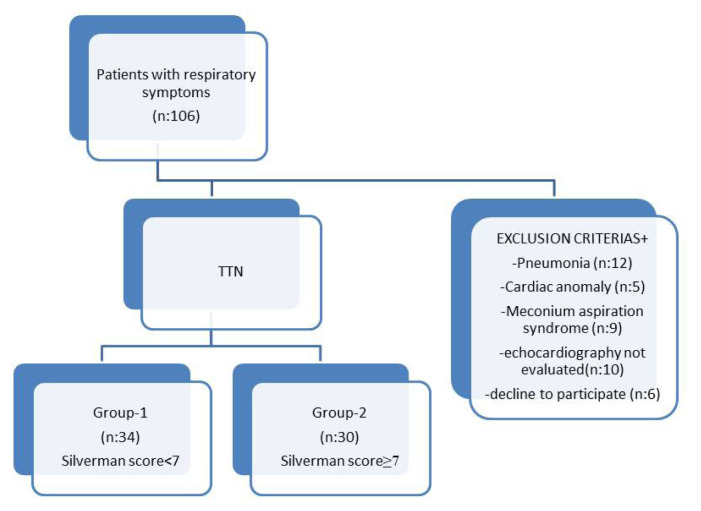
Study population and exclusion criteria.

**Table 1:** Comparison of the demographical features of the groups.

	Group 1 (n = 34)	Group 2 (n = 30)	p
	Silverman score < 7	Silverman score ≥ 7	

Sex, n (%)			
Female	12 (35.3)	14 (46.7)	0.355
Male	22 (64.7)	16 (53.3)	

Gestation week *	38 (34–42)	38 (35–41)	0.488

Birth weight + (gr)	3013.7 ± 344.3	3055.8 ± 390.1	0.648

Delivery mode, n (%)			
C/S	16 (47.1)	23 (76.7)	0.015
Vaginal	18 (52.9)	7 (23.3)	

Mother’s age+			
(years)	26.3 ± 5.4	26.4 ± 5.2	0.954

APGAR 1st min*	9 (7–9)	8 (6–9)	0.001

APGAR 5th min*	10 (8–10)	9 (8–10)	0.003

* Median (min–max)

+ Mean ± SD, C/S: cesarean section.

**Table 2:** The comparison of respiratory findings.

	Group 1 (n = 34)	Group 2 (n = 30)	p
	Silverman score < 7	Silverman score ≥ 7	

Respiratory rate at admission*	64 (60–78)	68 (62–88)	0.003

Downes score at admission*	4 (2–7)	7 (7–10)	0.001

Downes score at 24th h*	1 (0–3)	3 (1–10)	0.001

Silverman score at 24th h*	1 (0–3)	3 (1–9)	0.001

Oxygen free saturation at admission*	93 (84–98)	90 (81–94)	0.001

Duration of hospitalization*	3 (1–8)	6 (2–15)	0.001

Mode (%)			
Hood	25	3	0.001
nCPAP	9	17	
nSIMV	0	7	
SIMV	0	3	

Duration of oxygen treatment *	2 (1–5)	4 (1–14)	0.001

* Median (min–max),

nCPAP: nasal continuous positive airway pressure, nSIMV: nasal synchronized intermittent mandatory ventilation, SIMV: synchronized intermittent mandatory ventilation

**Table 3:** Comparison of hematologic parameters and RVSP of the groups.

	Group 1 (n = 34)	Group 2 (n = 30)	p
	Silverman score < 7	Silverman score ≥ 7	
White blood cell (WBC) (/mm^3^)+	17945.6 ± 5152.7	16159 ± 3787.6	0.123
Hemoglobin (Hb) (g/dL) +	18.4 ± 1.8	16.8 ± 1.6	0.01
Hematocrit (Hct) (%)+	55 ± 5.1	50.8 ± 5.0	0.01
MPV (fL) +	9.9 ± 0.8	10.2 ± 0.7	0.354
Platelet (/mm^)+	301500 ± 61222.5	336433 ± 50790	0.016
RDW (fL)+	65.7 ± 5.5	65.6 ± 6.9	0.937
Absolute NRBC count*	1.8 (0.2–12.8)	5.7 (0.6–31)	0.01
NRBCs/100 WBCs (%)*	0.38 (0.02–1.8)	1.0 (0.10–6.70)	0.01
Right ventricular systolic pressure (RVSP) (mmHg)*	17 (8–40)	24 (16–80)	0.001

* Median (min–max)

+ Mean ± SD,

MPV: mean platelet volume, NRBCs: nucleated red blood cells, RDW: red cell distribution width.

**Table 4:** Logistic regression analysis.

	OR	95% CI	p
Hemoglobin	0.383	0.197–0.745	0.005
APGAR 1st min	0.278	0.079–0.977	0.046
Respiratory rate at admission	1.314	1.012–1.705	0.040
RVSP	1.2	1.030–1.399	0.020
NRBCs/100 WBCs	7.065	1.258–39.670	0.026

RVSP: right ventricular systolic pressure, NRBCs: nucleated red blood cells.
